# Protective effects of niacin following high fat rich diet: an in-vivo and in-silico study

**DOI:** 10.1038/s41598-023-48566-8

**Published:** 2023-12-01

**Authors:** Noreen Samad, Natasha Manzoor, Ammara Batool, Aqsa Noor, Saima Khaliq, Sana Aurangzeb, Sheraz Ahmed Bhatti, Imran Imran

**Affiliations:** 1 Department of Biochemistry, Faculty of Science, Bahauddin Zakariya University, Multan, 60800 Pakistan; 2 Department of Biochemistry, Faculty of Science, Science and Technology, Federal Urdu University of Arts, Karachi, 75270 Pakistan; 3 Department of Biochemistry, Faculty of Science, University of Karachi, Karachi, 75270 Pakistan; 4 Department of Pathobiology, Faculty of Veterinary Science, Bahauddin Zakariya University, Multan, 60800 Pakistan; 5 Department of Pharmacology, Faculty of Pharmacy, Bahauddin Zakariya University, Multan, 60800 Pakistan

**Keywords:** Biochemistry, Computational biology and bioinformatics, Drug discovery, Neuroscience

## Abstract

Niacin had long been understood as an antioxidant. There were reports that high fat diet (HFD) may cause psychological and physical impairments. The present study was aimed to experience the effect of Niacin on % growth rate, cumulative food intake, motor activity and anxiety profile, redox status, 5-HT metabolism and brain histopathology in rats. Rats were administered with Niacin at a dose of 50 mg/ml/kg body weight for 4 weeks following normal diet (ND) and HFD. Behavioral tests were performed after 4 weeks. Animals were sacrificed to collect brain samples. Biochemical, neurochemical and histopathological studies were performed. HFD increased food intake and body weight. The exploratory activity was reduced and anxiety like behavior was observed in HFD treated animals. Activity of antioxidant enzymes was decreased while oxidative stress marker and serotonin metabolism in the brain of rat were increased in HFD treated animals than ND fed rats. Morphology of the brain was also altered by HFD administration. Conversely, Niacin treated animals decreased food intake and % growth rate, increased exploratory activity, produced anxiolytic effects, decreased oxidative stress and increased antioxidant enzyme and 5-HT levels following HFD. Morphology of brain is also normalized by the treatment of Niacin following HFD. In-silico studies showed that Niacin has a potential binding affinity with degradative enzyme of 5-HT i.e. monoamine oxidase (MAO) A and B with an energy of ~ − 4.5 and − 5.0 kcal/mol respectively. In conclusion, the present study showed that Niacin enhanced motor activity, produced anxiolytic effect, and reduced oxidative stress, appetite, growth rate, increased antioxidant enzymes and normalized serotonin system and brain morphology following HFD intake. In-silico studies suggested that increase 5-HT was associated with the binding of MAO with Niacin subsequentially an inhibition of the degradation of monoamine. It is suggested that Niacin has a great antioxidant potential and could be a good therapy for the treatment of HFD induced obesity.

## Introduction

Over the past few decades, sedentary lifestyle has promoted increase in intake of diet rich in lipids as a result substantial increase in body fat occurred, that is because of positive energy balance, favored by a high energy density diet such as high fat rich diet^1,2^. A high fat diet (HFD) is one, in which fats,both saturated and unsaturated, account for at least 35% of total calories consumed. Life style of People with a HFD (unhealthy) is possibly to develop obesity, and other chronic systemic diseases^3^. The consumption of HFD for the long term deregulates the normal functioning of various organs, including brain. Many evidences depict that it impairs the cognitive function and elevates the susceptibility to anxiety^3,4^ by formation of reactive oxygen species (ROS) and causes the oxidative stress and weakens the antioxidant mechanism in the brain^5^. However, the harmful effects of obesity in brain are still not clear, but previous findings had suggested that body fat deposition and obesity play an essential role in certain brain-associated disorders^6,7^ such as memory and learning dysfunction in the humans^8^ and in animal models^9^ and showed anxiety like behavior^10^ due to alteration in the levels of neurotransmitters and hormones which cause behavioral deficits^11^. Other diet such as ketogenic diet is also high in fat but the base of ketogenic diet is low dietary carbohydrate levels with variation in protein and fat contents. The ketogenic diet defined as a diet with 1gm/kg body weight,in which carbohydrates consumed as 10–15 gm/day and the outstanding energy taken from fat^12^. This diet produce ketosis, that can alter metabolic pathways to instigate weight loss and improve health conditions such as reduce blood glucose level and lipid profile^13^.

ROS elevated levels cause oxidative stress as a result of which cellular deterioration occurs^14^. An imbalance between antioxidant and ROS leads to oxidative stress. In the living system, the generation of ROS i.e., hydrogen peroxide (H_2_O_2_), superoxide and their by-products happen because of oxidative deterioration^15^. Brain is an organ which is very sensitive and highly vulnerable to the oxidative stress^16^. HFD has shown harm to the brain neurogenesis by elevating lipid peroxidation^17^. ROS oxidatively harm the biological molecules,on the other hand antioxidants such as glutathione peroxidase (GPx), catalase (CAT) and reduced glutathione (GSH) are consumed to counteract the free radicals^18^. Moreover, ROS production constantly occurs as an entailment of usual metabolic functions. If the antioxidant is weakened through an excess of ROS have been linked with some serious neurological diseases including anxiety, Alzheimer’s disease, etc.^19,20^.

The inhibitory effect of 5-HT on feeding is reported in animals and humans. The administration of drugs acting via serotonergic system affects appetite and fondness of fat^21^. In high HFD fed rats alteration in 5-HT neurotransmission was observed with reduced 5-HT-2C receptor mRNA expression in the hypothalamus of Osborne Mendel and S5BB/P1 rats^22^, enhanced 5-HT-2C and 5-HT-2A receptor binding density in the olfactory nucleus and hypothalamic nucleus in C57B1/6 obese mice^23,24^ and decreased 5-HT contents in Sprague Dawley rats^25^. Diet-induced obesity lowered the contents of 5-HT and its metabolite in the striatum^26^ and brain stem^25^ of rodents. Though, the mechanism on the serotonin system still remains unveiled.

Antioxidant augmentation is important in reducing oxidative stress and free radical production that reduce the obesity risk and associated problems. Vitamins are the organic molecules that are also important micronutrients that an organism requires in minute quantities for its proper metabolic functioning. Various vitamin supplements (Vit C, D, and B) show decreased anxiety in both animals and humans^27,28^. Both excess and deficiency of vitamins can cause significant illness, however the excess of water-soluble vitamins intake cannot do so. Niacin, also known as Nicotinic acid, is an organic molecule that is a type of vitamin B3. It plays a crucial role in keeping nervous system, and exhibiting antioxidant activity. Niacin contains two vitamers: Nicotinamide and Nicotinic acid. The body converts niacin to niacinamide, the most important kind of vitamin B3 in the blood^29^. Niacin has two enzymatic forms: NAD and NADP. Pellagra is caused by Niacin deficiency and symptoms include anxiety, memory loss, and headache^30^. Anxiolytic, antihyperlipidemic, antihypertensive, anti-inflammatory, hepatoprotective, and evident antioxidant properties all are found in Niacin. It is capable of scavenging ROS, because of its antioxidant, anti-inflammatory, and anti-apoptotic effects in many tissues and cells, Nicotinic acid is utilized to treat pathological conditions such as obesity^31^ and hyperalgesia^32^. Niacin is necessary for the survival and growth of the CNS. Evidence suggests that Niacin deficiency might cause disorders such as dementia, anxiety, sadness, and other symptoms comparable to those seen in neurodegenerative diseases^33^.

Though, many protective effects including antianxiety and enhanced motor activity of Niacin had reported^30^. Therefore the main aim of the present study was to investigate the neuroprotective effect of Niacin on behavioral, biochemical, neurochemical and histopathological parameters following HFD administration in rats. The in-silico study was further performed to monitoring the monoamine oxidase (MAO)-A and MAO-B activity of Niacin.

## Materials and methods

### Animals

Difference of hormones in male and female extensive studies have reported utilization of male rats in experimental studies^34^. In the present study, eight weeks old, male Sprague–Dawley rats with an average weight of 150–180 g were locally bred in the animal house of Department of Pharmacology, Bahauddin Zakariya University, Multan, Pakistan. Animals were kept in transparent cages, with selected diet. A 12/12-h light–dark cycle as well as a constant temperature of (21 ± 2 °C) was maintained. All experimental protocols were approved by bioethical committee of Department of Biochemistry, Bahauddin Zakariya University, Multan, Pakistan (D/1891/2021/Biochem,Dated 10/04/2021). The animals were treated ethically according to the strict guidelines of ‘Guide for the care and use of laboratory animals. It is stated that the study is reported in accordance with ARRIVE guidelines.

### Chemicals

Niacin, Reduced glutathione (GSH), sodium chloride, Di thio-bis-nitro benzoic acid (DNTB), trichloroacetic acid (TCA), 2-Thiobarbituric acid (TBA), Nitro blue tetrazolium (NBT), Hydroxylamine hydrochloride, H_2_O_2_, and other chemicals were purchased from Sigma Aldrich.

### Drug and diet preparation

Niacin was dissolved in water and given to animals via intraperitoneal (*ip*) injections for 8 weeks every day at the same time (9:00–10:00 am) with selected dose of 50 mg/ml/kg body weight of animal as reported previously^35^. Reported procedure was followed to prepare Normal diet (ND)^36^ and HFD^37^.

### Treatment schedule

Thirty-two rats were used in this study. Rats were divided into four groups. Each of the four groups had an equal number of animals (n = 8).

Group I: Water (1 ml/kg body weight) + ND.

Group II: Niacin (50 mg/ml/kg body weight) + ND.

Group III: Water (1 ml/ml/kg body weight) + HFD.

Group IV: Niacin (50 mg/ml/kg body weight) + HFD.

Both ND and HFD were weighed before placing in the cage. Food intake and body weight measured daily. The experiment continued for 2 month (56 days). Animals received their respective treatment daily. After eight weeks, behavioral test were performed. The light dark activity and elevated plus maze tests were used to assess anxiety-like behavior. The home cage and open field test were used to assess locomotor/exploratory activity. Behavioral activities were followed by decapitation without using rodent anesthesia as reported by Samad and Saleem^38^ and brains were removed and frozen at − 40 °C as done previously^39^ for biochemical examination such as superoxide dismutase (SOD), CAT, GPx, malondialdehyde (MDA), GSH, total protein content, and neurochemical estimation such as 5-HT and 5-hydroxyl indole acetic acid (5-HIAA) and histopathological studies.

### *%* Growth rate and food intake

Rats were weighed daily prior and thru the experiment. The *%* growth rate was deliberated by the formula:

$$\% {\text{growth rate}} = \, \left[ {{\text{Last}}\;{\text{day}}\;{\text{body}}\;{\text{weight}}/{\text{First}}\;{\text{day}}\;{\text{body}}\;{\text{weight}}} \right]*{1}00.$$

The diets weighed every day and positioned in hopper of home cage. Food intake (cumulative) saw by taking the variance between the food positioned on fist day and the food left after 24 h (till end day of the study).

### Behavioral analysis

#### Open field activity

The open field apparatus is used to determine rat exploratory/locomotor activity. The apparatus consists of an open novel space that is surrounded by a wall to prevent rats from escaping the field. The test was conducted in a quiet/novel environment with white light. The number of squares crossed along all four walls was counted for 5 min as reported earlier by Naqvi et al.^40^.

#### Home cage activity

A transparent home cage apparatus with specific dimensions was used to assess locomotor activity^41^. The home cage floor was covered with sawdust to provide the familiar environment to the animal. The activity (cage crossings) was recorded for 5 min.

#### Elevated plus maze (EPM) test

The apparatus was used to assess the anxiety like behavior as discussed by Naqvi et al.^40^. The apparatus was consisted of four arms, two of which were closed and the other two were opened. The elevated plus maze is fixed at 60 cm above the ground. Rats were placed in the central space allowing animal to observe the surroundings. The time rats spent in open arms was recorded. Each rat in the experiment was given 5 min to spend in the apparatus.

#### Light/dark activity (LDA) test

The experiment was carried out in chambers having two parts. Those parts are equal in length that is (26 × 26 × 26 cm) and an opening of (12 × 12 cm) connects the 2 parts. The light part was translucent allowing light to pass through the walls. The other part was dark and opaque preventing light from passing through. Rats were placed in the dark compartment and the time rats spent in the light part was noted for 5 min as reported previously^38,42^.

### Biochemical analysis

After decapitation, behavioral tests were conducted. All animals' brains were removed, and washed with normal saline solution (0.9%) before being utilized for various biochemical analysis. For biochemical analysis, a 10% (wt.\vol.) brain homogenate was combined with 0.1 M, (pH 7.4) phosphate buffer and centrifuged for 20 min at 4 °C.

#### Determination of malondialdehyde (MDA)

Estimation of MDA content in the brain was performed same as reported earlier^42^. In a test tube, 2 ml of TCA-TBA and 3 ml of brain homogenate were combined. This mixture was heated in a water bath for around 15 min. The mixture was allowed to cool. The next step was to centrifuge it for 10 min at 3500 rpm. The absorbance was recorded at 532 nm using supernatant.

#### Determination of superoxide dismutase (SOD)

Estimation of SOD was performed according to the procedure mentioned previously^43^. 0.5 ml homogenate of brain tissue (10%) from each sample was treated with 1 ml NaHCO_3_, 0.2 ml EDTA (0.6 mM). and 1.0 ml carbonate–bicarbonate (0.1 M,pH 10.2) buffer. The reaction was started by adding 0.4 ml NBT and the absorbance was recorded at 560 nm. The % inhibition was calculated using a blank as a standard.

#### Determination of catalase (CAT)

To calculate the activity of CAT already reported procedure was followed^44^. To make the filtrate, 0.1 ml of brain tissue homogenate (10%) in 0.01 M phosphate buffer (pH 7.0) was added. After that 0.1 ml of filtrate was mixed with 1.4 ml of reaction mixture that contained 0.4 ml of 2 M H_2_O_2_ and 1 ml of same phosphate buffer. After 1 min reaction was terminated by adding dichromate-acetic acid reagent. Distilled water was used at the place of filtrate as Blank. The % inhibition of CAT was determined by measuring the absorbance at 620 nm.

#### Determination of glutathione peroxidase (GPx)

To calculate activity of GPx, already reported procedure of Samad et al.^42^ was used. To prepare 1 ml of reaction mixture,0.1 ml NaNO_3_, 0.2 ml GSH (2 mM), 0.3 ml brain supernatant, 0.3 ml of phosphate buffer (0.1 M, pH 7.4) and, 0.1 ml of H_2_O_2_ were added to the test tubes. After that, the reaction mixture was incubated at 37 °C for 15 min. In each test tube, 0.5 ml of 10% TCA was added. The supernatant was collected after centrifugation at 15,000 rpm for 5 min. 0.7 ml (4 mg/ml) of DTNB, 0.2 ml (0.1 M, pH 7.4) of phosphate buffer, and 0.1 ml of brain supernatant were mixed in test tube. At 420 nm, the absorbance was measured.

#### Determination of reduced glutathione (GSH)

GSH was calculated when it reacted with Ellman's reagent, and the yield of this reaction was yellow chromophore^45^. Brain homogenate (10%) and 15% Per chloric acid were taken in equal parts and stored at 4 °C for one night. Per chloric acid treated samples were centrifuged at 2000 rpm for 10 min. The supernatant, 0.4 ml of distilled water, 2 ml of phosphate buffer (pH 8.4), 0.1 ml of tissue sample, and 0.5 ml of DTNB were mixed to estimate GSH. At 412 nm, the absorbance was measured, and levels of GSH were calculated.

#### Determination of total protein activity

To determine protein contents, already reported procedure was followed^46^. 1 ml of pallet from GSH activity was mixed with 0.1 N sodium hydroxide and let aside for 10 min, then 0.5 ml of Folin reagent was mixed and set away for another 10 min. At 610 nm, the absorbance was measured. Bovine standard solution (200 mg in 100 ml distilled water) was used to assess total protein levels.

### Neurochemical estimation

The evaluation of 5-HT and 5-HIAA contents in the rat’s brain performed by HPLC-EC. The analysis was done as described earlier^47^.

### Histopathological estimation

The brain was kept static in a 10% formalin solution for histological analysis. After sometime brain was placed in paraffin wax as previously described by Thenmozhi et al.^48^. 5 µm thick piece of brain was stained with hemoxylin and eosin. A light microscope set to 400 × was used to monitored the changes in brain tissue sections.

### In-silico analysis of niacin

#### The procedure of homology modeling

The sequences of the rat target protein, i.e., MAO-A, and MAO-B, were retrieved from Uniprot Knowledgebase (http://www.uniprot.org/). The sequence lengths of these proteins consist of 526, and 520 amino acids respectively. The potential templates were identified by running the Psi-BLAST tool^49^ to screen the protein data bank PDB (https://www.rcsb.org)^50^. The following crucial considerations went into selecting the best templates, i.e., highest sequence identity, maximum coverage, fewer gaps, and minimum E-value. The secondary structure information of all these proteins was retrieved from PSI-blast based secondary structure PREDiction (PSIPRED) to improve the quality of alignments between the targets and the templates^51^. For the prediction of the best quality model, the missing residues from the selected templates were repaired by Modeller v.10.2^52^. Using the optimized sequences of the targets and templates as input, the homology modeling was performed by the modeler. This structure prediction was carried out for protein, MAO-B whereas the MAO-A crystal structure of rat was available at PDB^50^. The quality of the predicted models was evaluated by two standalone software, i.e., Procheck^53^ and Prosa^54^. The output of Procheck was Ramachandran plots which showed the stereochemistry of the predicted models, and the output of Prosa was energy plots which pointed to the potential error in the predicted models by computing the overall energy score. The molecular modeling software, i.e., DS visualizer v.4.5^55^ and Pymol v.2.5.0^56^, were used to analyze the active binding site and overall three-dimensional architectures of the predicted models.

#### The procedure of molecular docking

To analyze the In-silico effect of Niacin against rat proteins, i.e., MAO-A, and MAO-B, molecular docking studies were carried out. Autodock Vina v.1.1.2^57^ is the protein–ligand docking software that was utilized because of having extraordinary qualities such as high speed, accurate docking, better scoring function, and efficient optimization. The three-dimensional ligand files for docking studies were prepared using the Marvin Sketch v.16.2.8^58^ and saved in mol format. Autodock tools (ADT) v.1.5.7^59^ was used to assign the Gasteiger charges, computation of torsion angles, and saved in pdbqt format of these ligand files. The files of the receptors were also prepared for docking studies by using the ADT by the addition of polar hydrogen atoms, assignment of Kollman charges as well as assuring and repairing the missing atoms in the predicted model of rat proteins. For docking, the grid boxes were also set using the ADT. The grid box highlights the specific area that is used for docking calculations,hence the dimensions of the grid boxes were set differently for distinct receptors. The monoamine oxidase A grid box was set to 12 × 12 × 12, x, y, z, coordinates = 44.346, 12.574, 149.384 with a grid spacing of 1 Å. The monoamine oxidase B grid box was set to 30 × 30 × 30, x, y, z, coordinates = 51.543, 158.112, 20.414 with a grid spacing of 1 Å. The exhaustiveness value was equal to 8 and was the same for the receptors. In the end, all the receptor files were saved in pdbqt format. DS Visualizer v.4.5^55^, Ligplot v.2.2.5^60^, and Pymol v.2.5.0^56^ were used to create the two-dimensional and three-dimensional interactions images of the docked complexes.

### Statistical analysis

Tukey's test and 2-way ANOVA were used to assess all of the behavioral and biochemical data. SPSS version 20 was used to estimate the analysis (IBM, Chicago, IL, USA). *p* < 0.05 was used to indicate a significant value (Table 1).

**Table 1:** Tabulation form of statistical analysis by Two-way ANOVA.

Results	df	Effect of Niacin Treatment	Effect of HFD	Interaction between Niacin*HFD
% Growth rate	1,28	F = 419.43, *p* < 0.05	F = 541.61, *p* < 0.05	F = 197.90, *p* < 0.05
Food intake	1,28	F = 90.73, *p* < 0.05	F = 238.25, *p* < 0.05	F = 53.39, *p* < 0.05
Open field test	1,28	F = 215.13, *p* < 0.05	F = 669.69, *p* < 0.05	F = 59.37, *p* < 0.05
Home cage activity	1,28	F = 490.83, *p* < 0.05	F = 196.67, *p* < 0.05	F = 4.12, *p* < 0.05
Light dark activity box	1,28	F = 79.28, *p* < 0.05	F = 33.50, *p* < 0.05	F = 88.43 *p* < 0.05
Elevated plus maze test	1,28	F = 44.26, *p* < 0.05	F = 421.25, *p* < 0.05	F = 14.26, *p* < 0.05
GSH	1,28	F = 430.27, *p* < 0.05	F = 150.58, *p* < 0.05	F = 26.35, *p* < 0.05
Total protein contents	1,28	F = 85.331, *p* < 0.05	F = 189.92, *p* < 0.05	F = 6.53, *p* < 0.05
MDA	1,28	F = 32.33, *p* < 0.05	F = 89.95, *p* < 0.05	F = 5.62, *p* < 0.05
GPx	1,28	F = 120.82, *p* < 0.05	F = 158.83, *p* < 0.05	F = 15.48, *p* < 0.05
CAT	1,28	F = 220.53, *p* < 0.05	F = 450.29, *p* < 0.05	F = 5.8, *p* < 0.05
SOD	1,28	F = 162.28, *p* < 0.05	F = 428.76, *p* < 0.05	F = 10.53, *p* < 0.05
5-HT	1,28	F = 146.25, *p* < 0.05	F = 586.78, *p* < 0.05	F = 9.48, *p* < 0.05
5-HIAA	1,28	F = 136.25. *p* < 0.05	F = 385.42, *p* < 0.05	F = 12.45, *p* < 0.05

### Ethical approval

Institutional Bio-ethical committee ((D/1891/2021/Biochem; Dated 10/04/2021) was received for the animal experiment from the Department of Biochemistry, Bahauddin Zakariya University, Multan, Pakistan.

## Results

### Effect of niacin on cumulative food intake and body weight changes in ND and HFD fed rats

**Figure 1:**
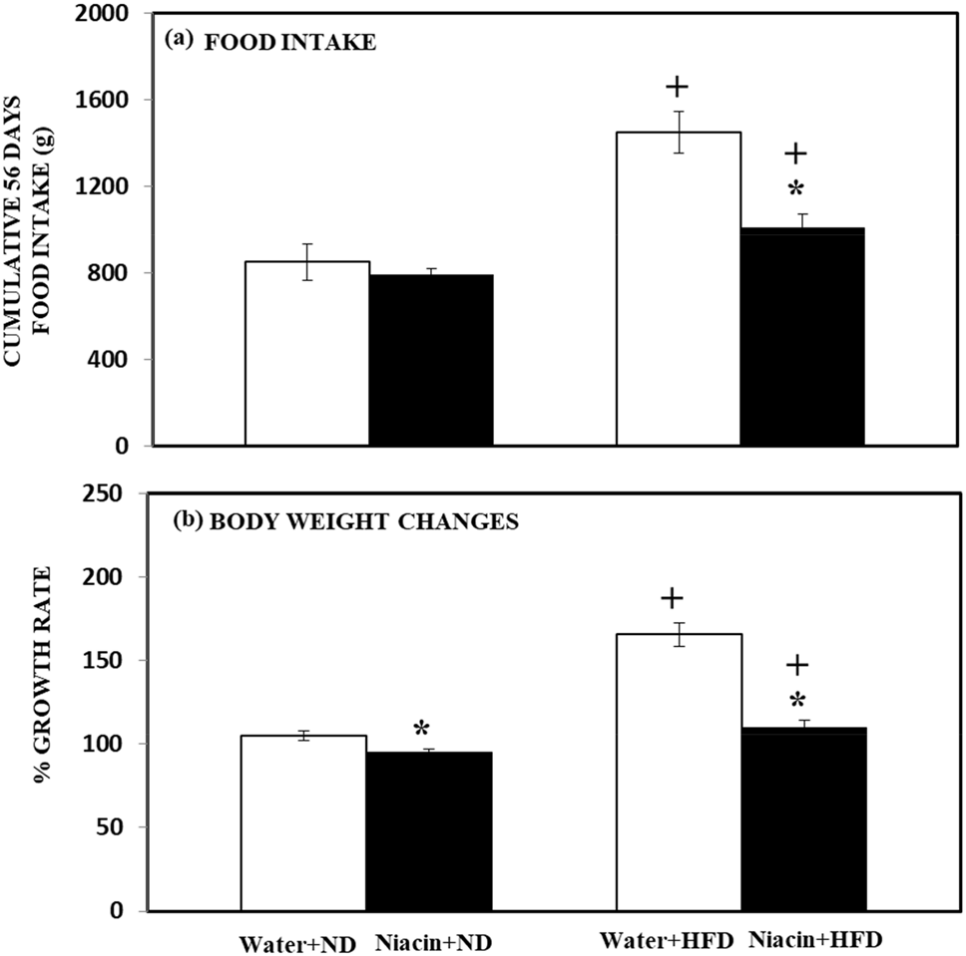
Effect of Niacin in food intake and %growth rate. Mean of values is ± S.D (n = 8). 2-way ANOVA was used to investigate the data. Notable substantial alterations are monitored through Tukey 's test *p < 0.05 between ND and HFD treated rats.

HFD increased (*p* < 0.05) food intake in both control and Niacin treated animals. The administration of Niacin decreased (*p* < 0.05) food intake in HFD than ND fed rats (Fig. 1a).

HFD increased % growth rate in both control and Niacin treated animals. The administration of Niacin decreased % growth rate in HFD and ND fed rats (Fig. 1b).

### Effect of niacin on exploratory activity (open field and home cage activity) in ND and HFD fed rats

**Figure 2:**
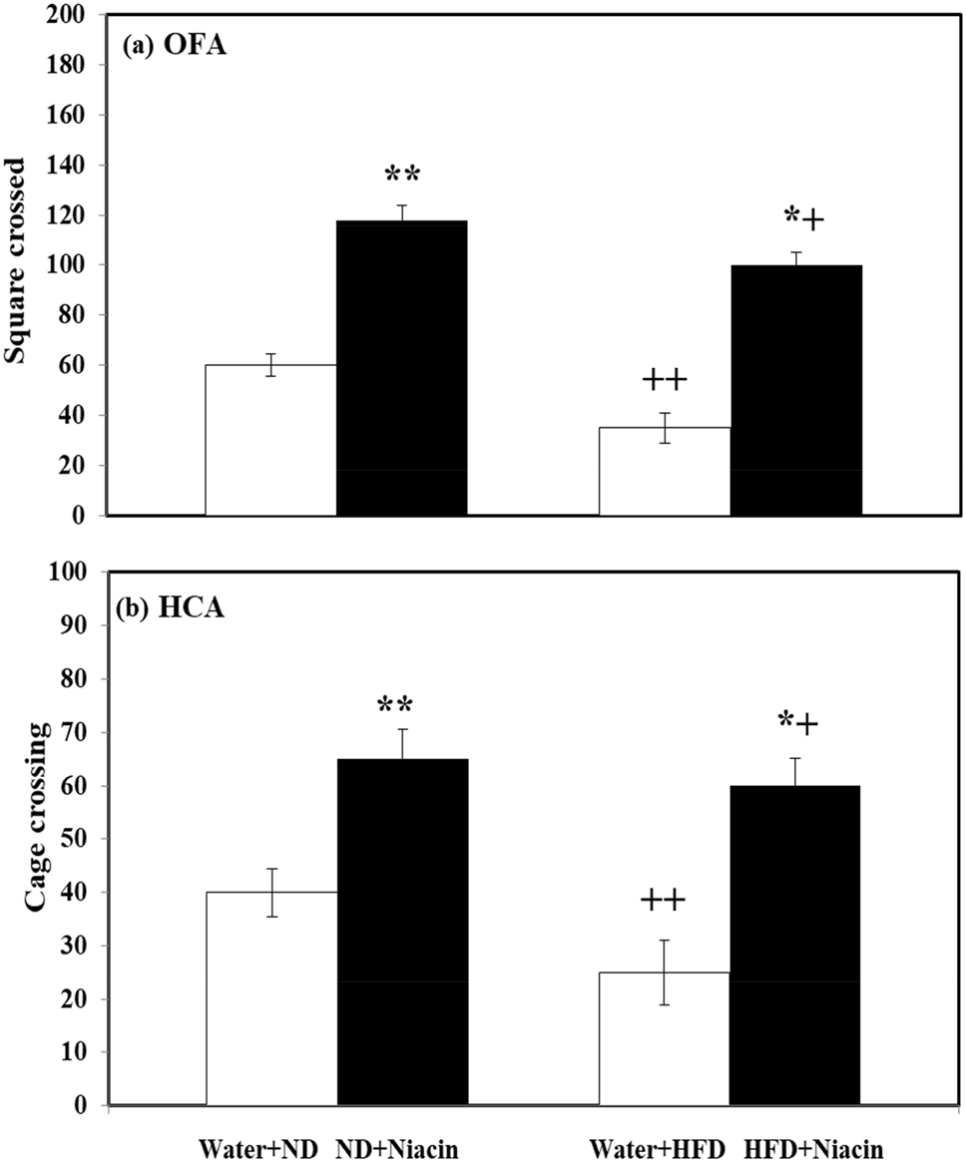
Effect of Niacin in open field (**a**) and home cage (**b**) activity. Mean of values is ± S.D (n = 8). 2-way ANOVA was used to investigate the data. Notable substantial alterations are monitored through Tukey 's test *p < 0.05 between ND and HFD treated rats.

Niacin increased (square crossed in open field activity test in both ND (*p* < 0.01) and HFD (*p* < 0.05) fed animals. HFD fed rats exhibited smaller square crossed in water (*p* < 0.01) and Niacin (*p* < 0.05) treated than their respective control (Fig. 2a).

Niacin increased cage crossings in home cage activity in both ND (*p* < 0.01) and HFD (*p* < 0.05) fed animals. HFD fed rats exhibited smaller cage crossings in water (*p* < 0.01) and Niacin (*p* < 0.05) treated than their respective control (Fig. 2b).

### Effect of niacin on anxiety profile in ND and HFD fed rats

**Figure 3:**
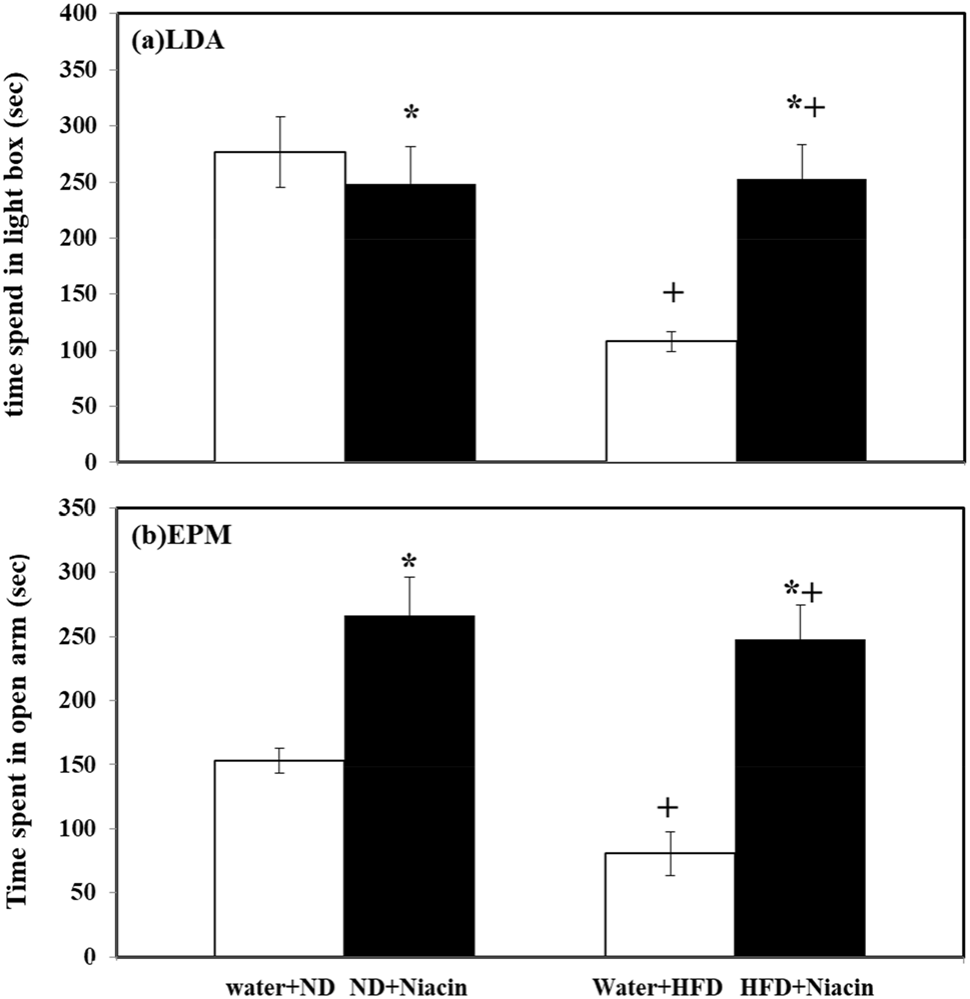
Effect of Niacin in light dark box (**a**) and elevated plus maze (**b**) activity. Mean of values is ± S.D (n = 5). Data are investigated via 2-way ANOVA. Notable substantial alterations are monitored through Tukey’s test *p < 0.05 between ND and HFD treated rats.

Niacin decreased (*p* < 0.05) and increased (*p* < 0.05) time spent in light compartment in ND and HFD fed rats respectively. HFD fed rats exhibited less (*p* < 0.05) time spent in both control and Niacin (Fig. 3a).

Niacin increased time spent in open arm in in both ND (*p* < 0.05) and HFD (*p* < 0.05) fed animals. HFD fed rats exhibited less time spent in both control and Niacin treated than their respective control (Fig. 3b).

### Effect of niacin on brain total protein and GSH contents in ND and HFD fed rats

**Figure 4:**
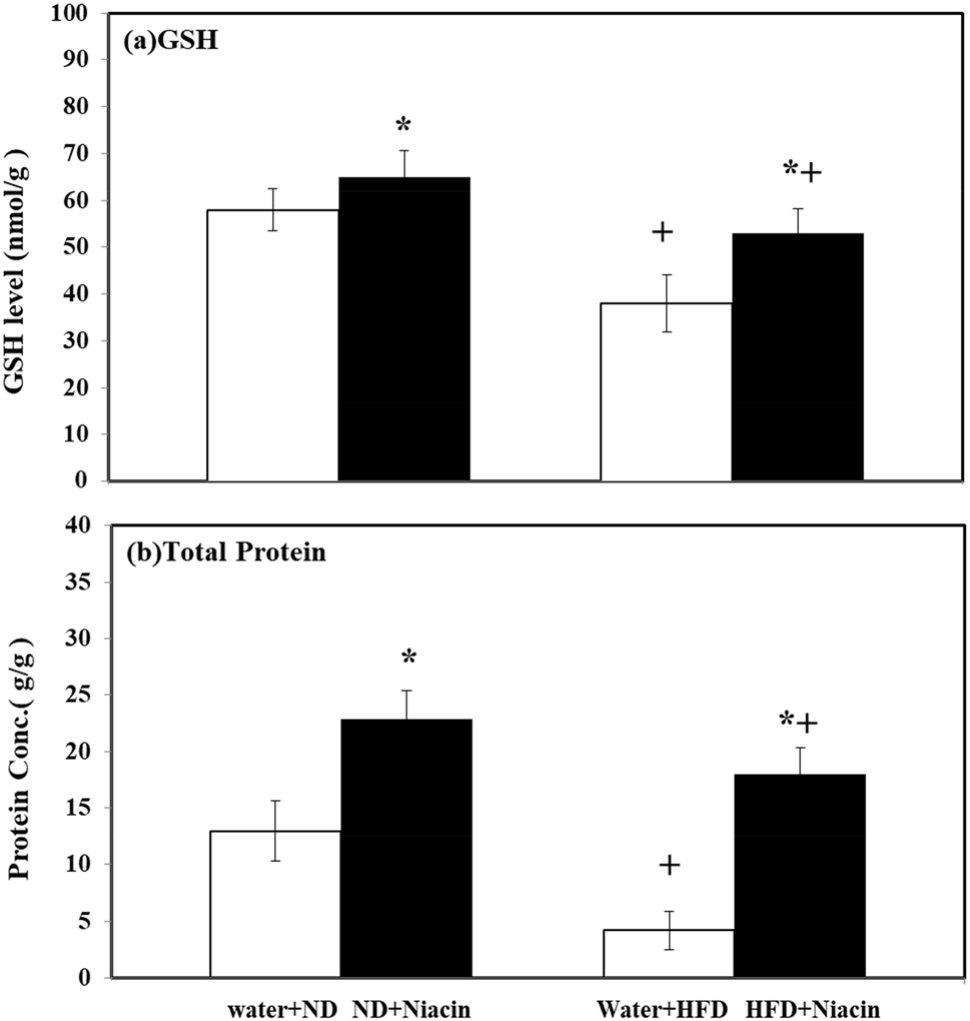
Effect of Niacin on GSH (**a**) and total protein content (**b**) in brain of the rats. Mean of the values is ± S.D (n = 8). 2-way ANOVA was used to investigate the data. Notable substantial alterations are monitored via Tukey 's test *p < 0.05 between ND and HFD treated rats.

Niacin increased GSH contents in both ND and HFD fed animals. HFD fed rats exhibited reduced GSH contents in both control and Niacin treated than their respective control (Fig. 4a).

Niacin increased protein contents in both ND and HFD fed animals. HFD fed rats exhibited reduced protein contents in both control and Niacin treated than their respective control (Fig. 4b).

### Effect of niacin on brain oxidative stress in ND and HFD fed rats

**Figure 5:**
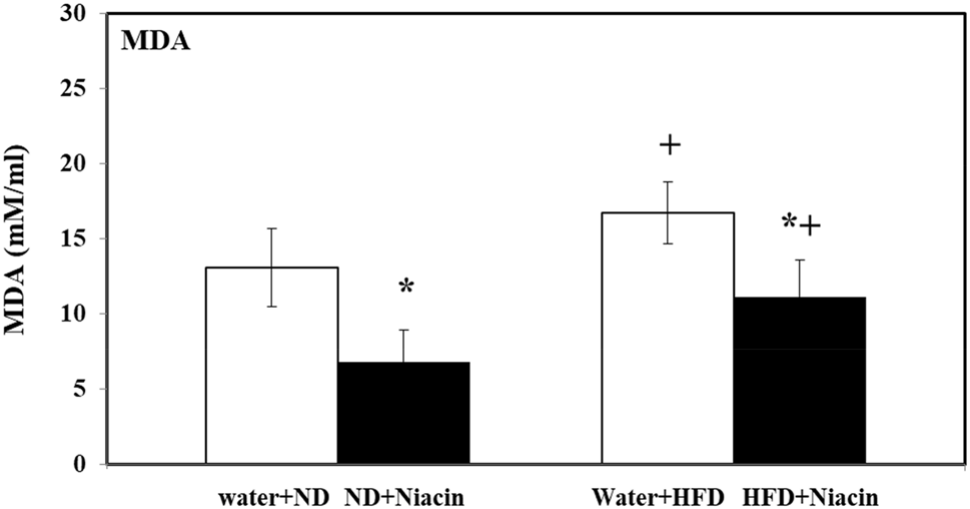
Effect of Niacin administration on lipid peroxidation in rat brain. Mean of the values is ± S.D (n = 8). 2-way ANOVA was used to check the data. Notable substantial alterations are monitored via Tukey’s test *p < 0.05 between ND and HFD treated rats.

Niacin decreased MDA contents in both ND and HFD fed animals. HFD fed rats exhibited elevated MDA contents in both control and Niacin treated than their respective control (Fig. 5).

### Effect of niacin on brain antioxidant enzymes in ND and HFD fed rats

**Figure 6:**
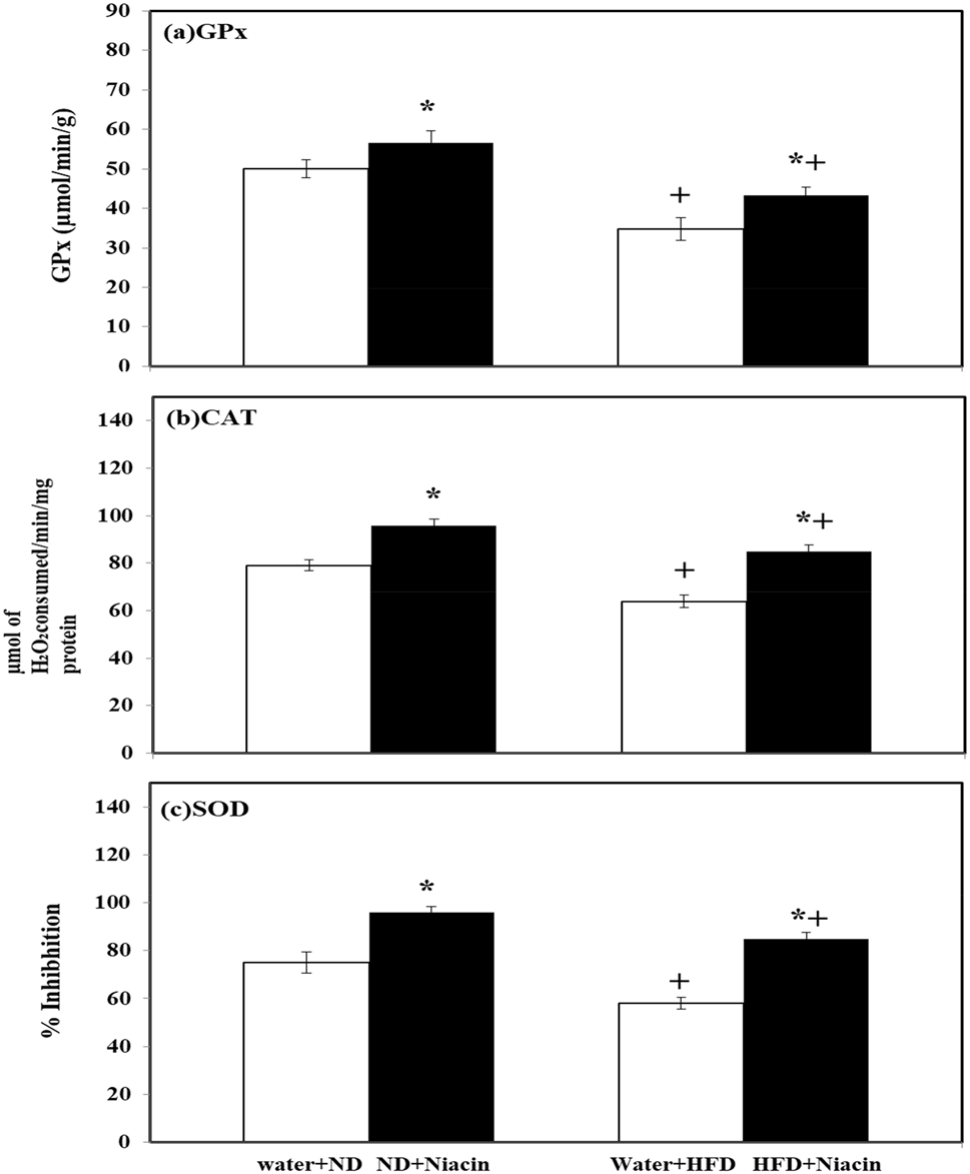
Effects of Niacin administration on brain GPx (**a**), CAT (**b**) and SOD (**c**) activity. Mean of the values is ± S.D (n = 8). 2-way ANOVA was used to check the data. Notable substantial alterations are monitored via Tukey 's test *p < 0.05 between ND and HFD treated rats.

Niacin increased activity of GPx in both ND and HFD fed animals. HFD fed rats exhibited decreased activity of GPx in both control and Niacin treated than their respective control (Fig. 6a).

Niacin increased activity of CAT in both ND and HFD fed animals. HFD fed rats exhibited decreased activity of CAT in both control and Niacin treated than their respective control (Fig. 6b).

Niacin increased SOD activity in both ND and HFD fed animals. HFD fed rats exhibited decreased SOD activity in both control and Niacin treated than their respective control (Fig. 6c).

### Effect of niacin on brain serotonin metabolism in ND and HFD fed rats

**Figure 7:**
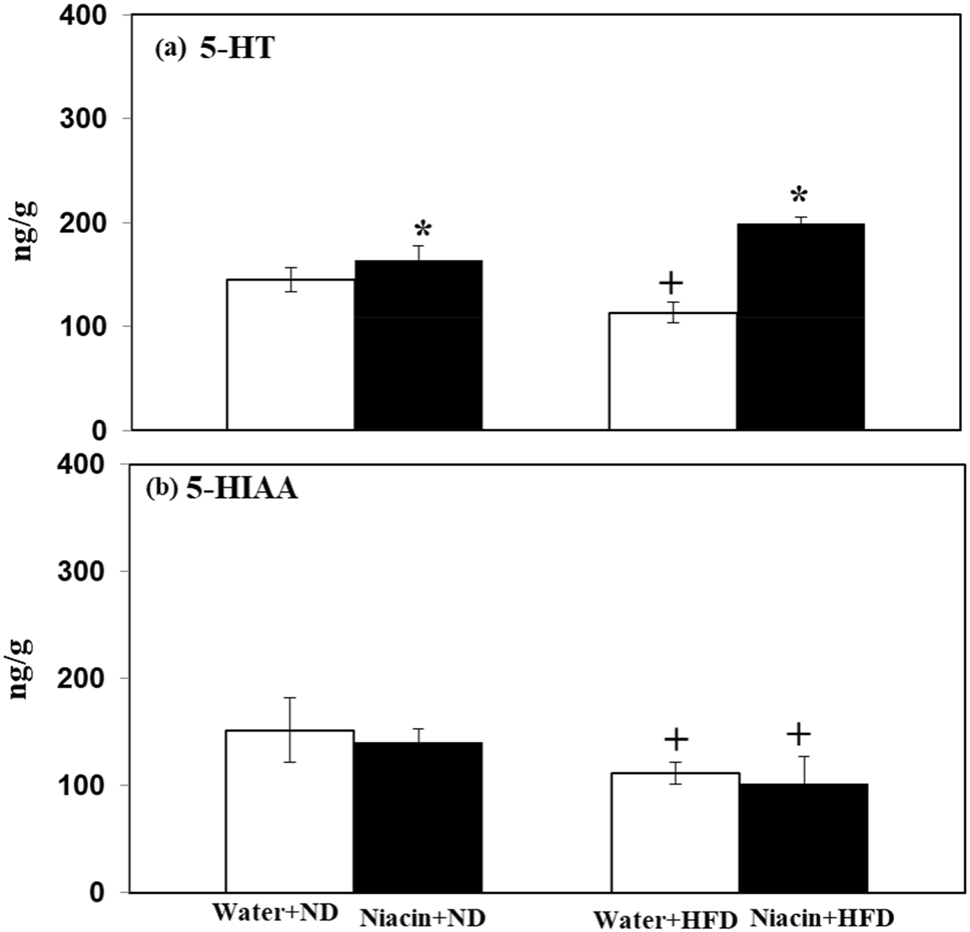
Effects of Niacin administration on brain 5-HT (**a**) and 5-HIAA (**b**) levels in rat brain Mean of the values is ± S.D (n = 8). 2-way ANOVA was used to check the data. Notable substantial alterations are monitored via Tukey’s test *p < 0.05 between ND and HFD treated rats.

Niacin increased 5-HT levels in both ND and HFD fed animals. HFD fed rats exhibited decreased levels of 5-HT control rats (Fig. 7a).

HFD fed rats exhibited decreased levels of 5-HIAA in both control and Niacin treated rats than their respective control (Fig. 7b).

### Molecular docking

Niacin substantially induced anxiolytic behavior and increased serotonin metabolism in ND and HFD fed animals. We performed molecular docking to predict interactions of Niacin with MAO-A and B to evaluate its binding affinity and selectivity against MAO-A and MAO-B as therapeutic agents for psychiatric illnesses.

#### Monoamine oxidase (MAO)

**Figure 8:**
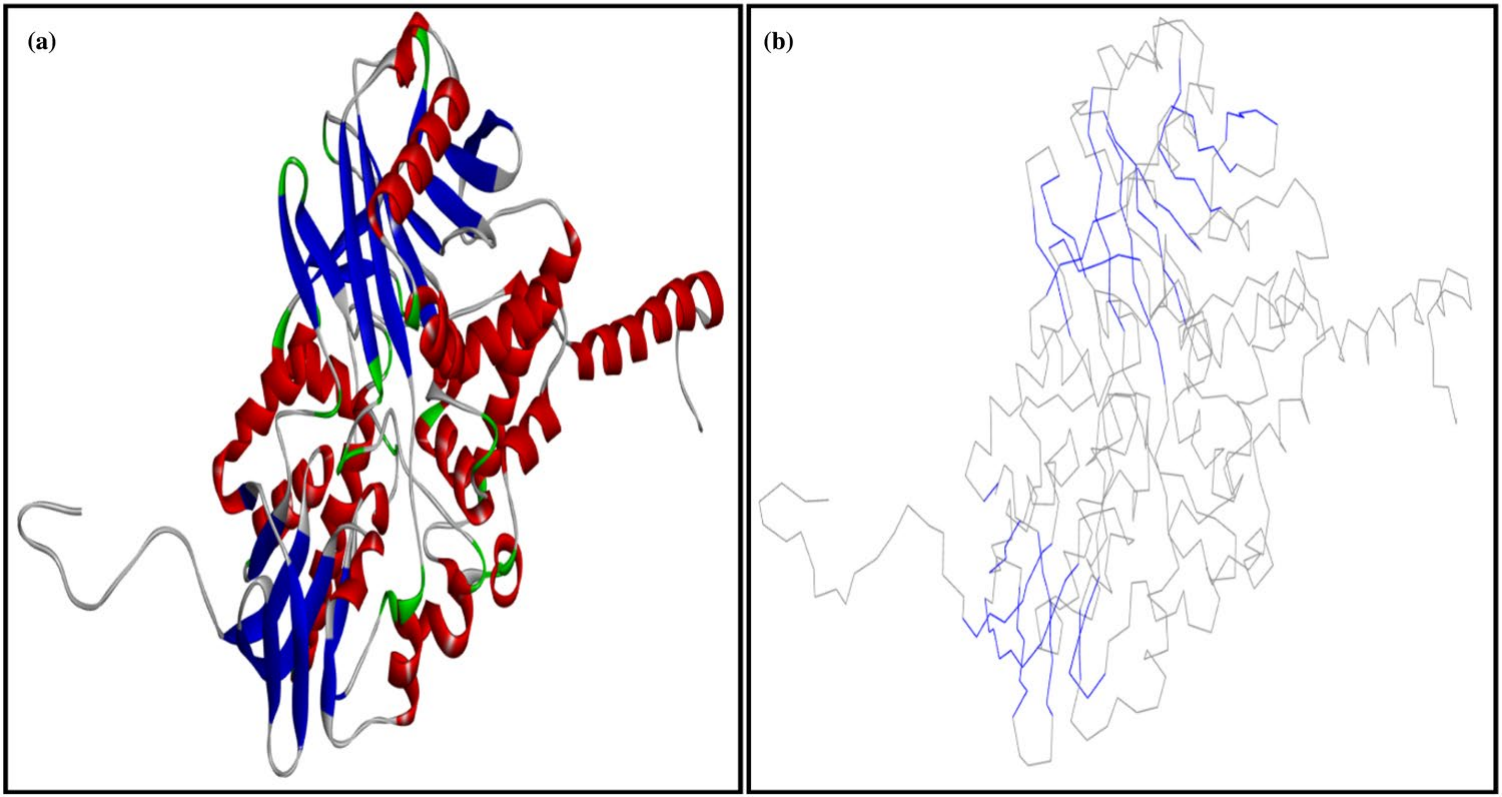
(**a**) crystal structure of rat monoamine oxidase A (PDB ID: 1O5W), (**b**) crystal structure of rat monoamine oxidase A, which was colored according to C-alpha atoms.

**Figure 9:**
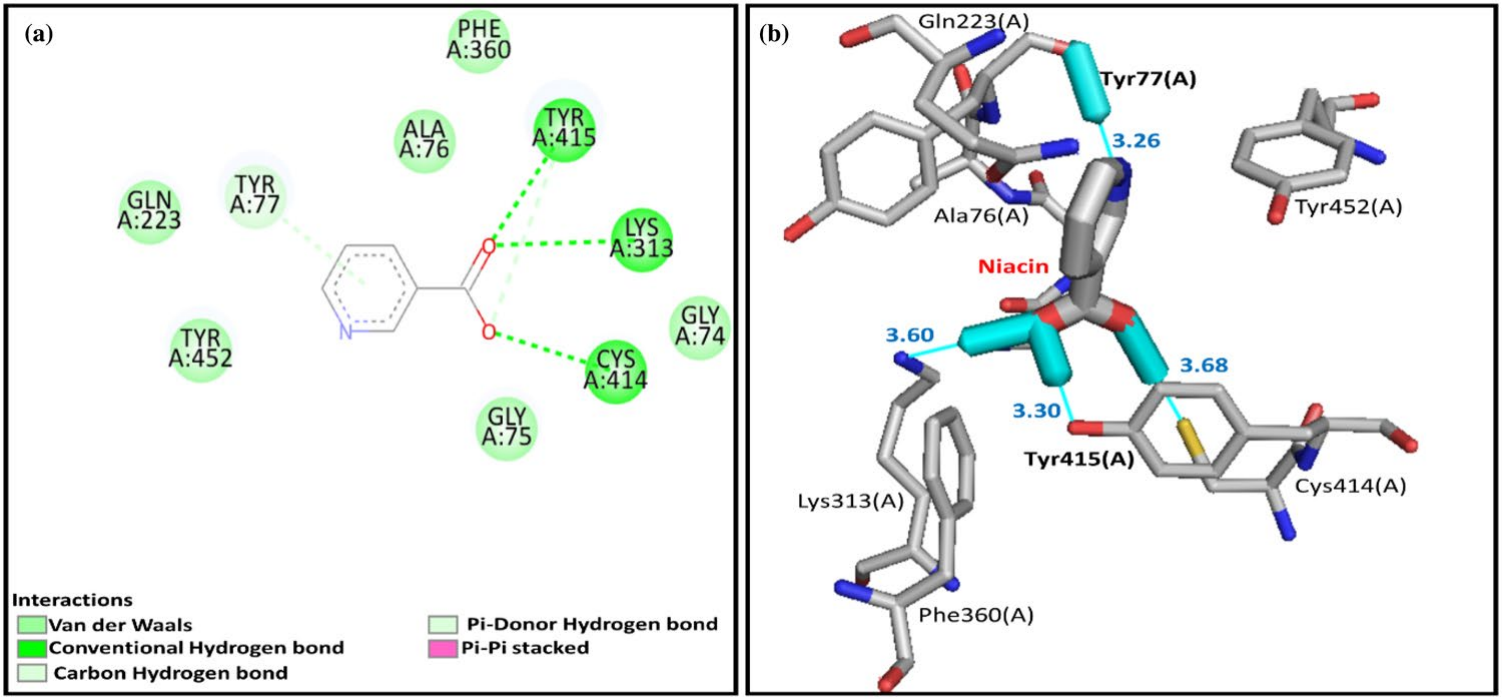
(**a**) two-dimensional and (**b**) three-dimensional interactions between niacin and active residues of rat monoamine oxidase A.

The MAO is responsible to regulate the crucial activities of the central nervous system. It is a mitochondrial outer-membrane-bound flavoenzyme and is also responsible for the catalysis of several important neurotransmitters such as serotonin, norepinephrine, and dopamine^61^. There are two types of MAO, i.e., MAO-A and MAO-B. Both enzymes share approximately 70% sequence identity and differ from each other as having unique substrate and inhibitor specificities^62^. Structurally MAO-A is a dimeric structure (Fig. 8), and the overall folding of the dimeric structure is immensely similar^63^. Each monomer of the MAO-A structure is further divided into three domains, i.e., the FAD binding domain, the substrate binding domain, and the membrane binding domain^64^. The site-directed mutagenesis studies revealed the MAO-A active amino acids, i.e., K305, W397, Y407, and Y444. These residues are involved in non-covalent interactions with FAD. The aromatic residues Y407 and Y444 may form a sandwich that has stabilized the substrate binding^65^. Here, missing residues were repaired in the crystal structure of the rat MAO-A, so the positions of these active residues become K313, W405, Y415, and Y452 (Fig. 9).

#### Predicted binding mode of niacin in the active pocket of the MAO-A

**Figure 10:**
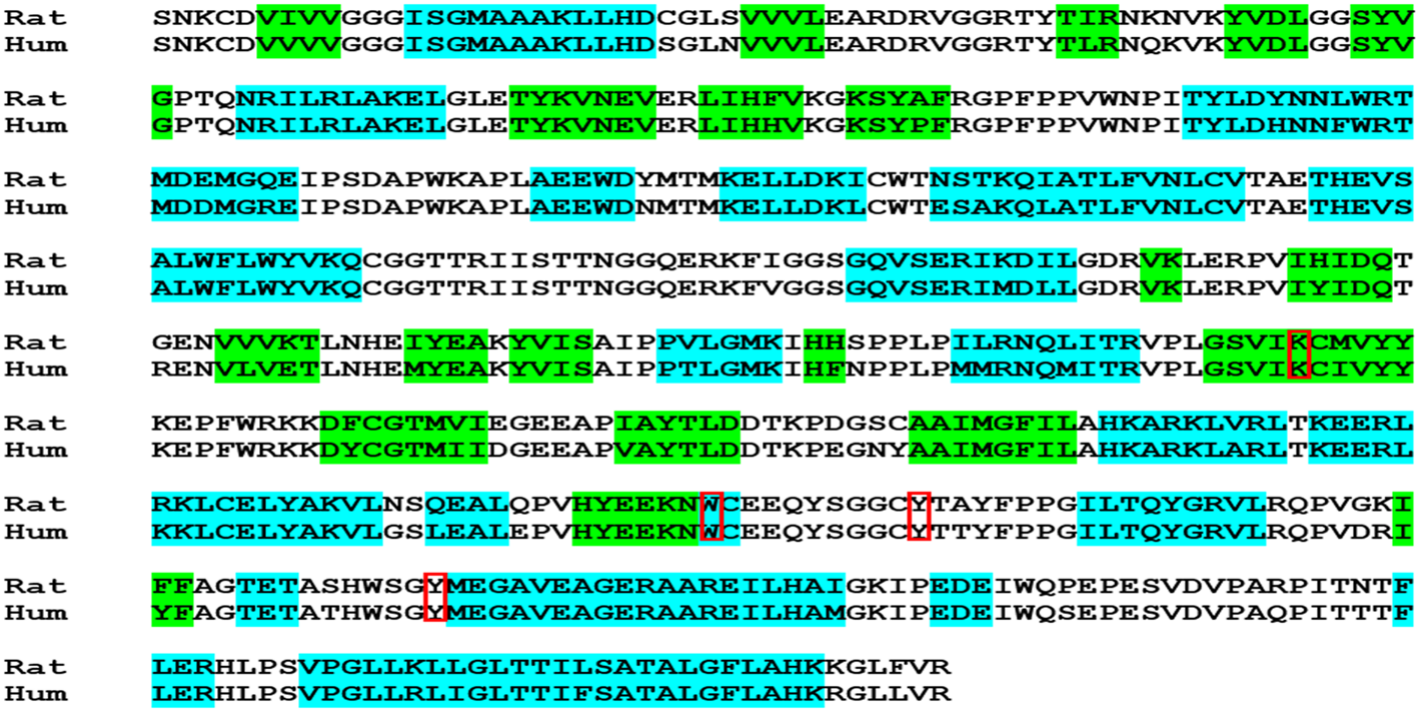
pair-wise sequence alignment between the human monoamine oxidase (PDB ID: 2XFU) and the rat monoamine oxidase A (UniProt Accession Number: P19643). The light blue highlighted residues were involved in making α-helices, while the green highlighted residues formed β-sheets, the predicted secondary structure. The active site residues are in red boxes.

The binding affinity between the Niacin and the active site of MAO-A was − 4.5 kcal/mol. The interaction analysis between Niacin and the active pocket residues of MAO-A active residues depicted that the niacin made four hydrogen bonds with Y77, C414, K313, and Y415, and the lengths of these hydrogen bonds were 3.26 Å, 3.68 Å, 3.60 Å, and 3.30 Å respectively (Fig. 9a and b). The aromatic residue, i.e., Y77, made a π-donor hydrogen bond (Fig. 9a), and the other active residues, i.e., G74, G75, A76, Q223, and Y452, made van der Waals interactions with the niacin and were stabilized the docked complex. In conclusion, the main active residues, i.e., K313, Y415, and Y452, made significant interactions with the Niacin and revealed accurate docking of niacin in the active pocket of the MAO-A with good affinity. Pair-wise sequence alignment between the human monoamine oxidase (PDB ID: 2XFU) and the rat monoamine oxidase A (Fig. 10).

#### Homology modeling studies of the MAO-B

**Figure 11:**
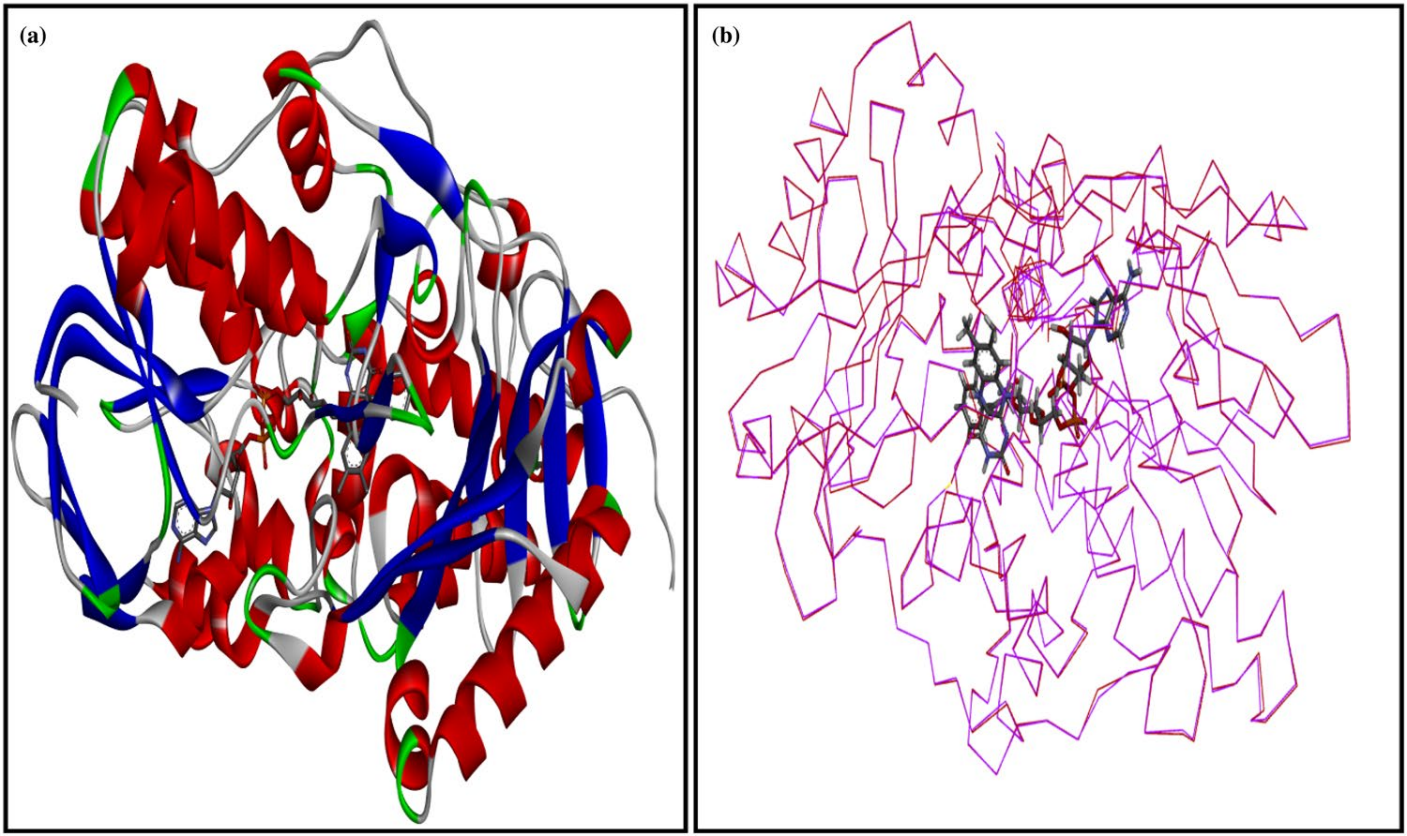
(**a**) predicted model of rat monoamine oxidase B, (**b**) superimpose structure of rat and human monoamine oxidase B complexed with tranylcypromine (inhibitor).

**Figure 12:**
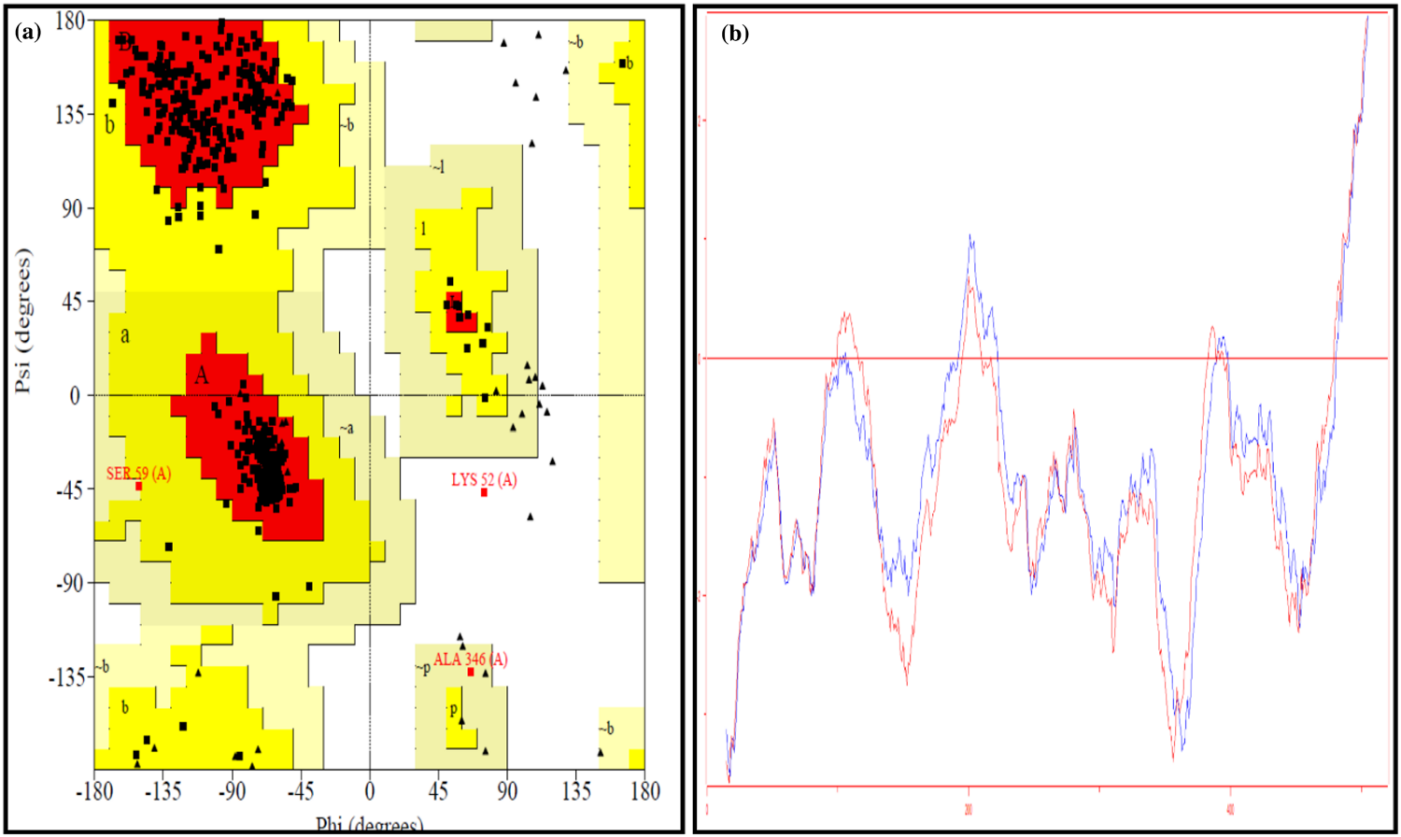
(**a**) Ramachandran plot shows that one residue in the disallowed region and 93.9% residues in the most favored region, (**b**) energy plot of rat monoamine oxidase is blue, and the energy plot of human monoamine oxidase is red.

The rat MAO-B homology model was based on the crystal structure of human monoamine oxidase B (Fig. 11a). The predicted and the crystal structures shared 89% sequence similarity (Fig. 12). Model evaluation by Ramachandran and energy plots showed the good quality of the predicted model. The Ramachandran plot analysis illustrated that there were 93.9% residues in the most favored region, and only one residue, i.e., Lys 52, was evident in the disallowed region. The polar residue, i.e., K52, is the residue that was found in the disallowed region of the crystal structure also (Fig. 12a), as this residue is not part of the regular secondary structure as well as not part of the active site which showed its unaffectedness on the predicted model quality. The energy plot also presented a satisfactory profile with similar peaks and indicated no potential errors in the predicted model (Fig. 11b). The structural investigations on MAO-B revealed that it is a dimeric structure^63^. Each monomer of the MAO-B structure is further divided into three domains, i.e., the FAD binding domain, the substrate binding domain, and the membrane binding domain^64^. The computed root-mean-square-deviation (r.m.s.d) between the predicted homology model and the crystal structure was much lower, i.e., 0.29 Å that revealed a similar three-dimensional fold (Fig. 12b). The site-directed mutagenesis studies demonstrated that the MAO-B active amino acids are K295, W387, Y397, and Y434, and these residues are involved in non-covalent interactions with Flavin adenine dinucleotide. The aromatic residues, i.e., Y398 and Y435, may form a sandwich and be stabilized the substrate binding^65^. The positions of these active residues in predicted structures were K296, W388, Y398, and Y435. These residues are in red boxes in Fig. 12. The imidazoline compounds were reported as potent hypertensive agents and the binding site of these compounds in human MAO-B is different revealed by crystallographic and biochemical studies^66^.

#### Predicted binding mode of niacin in the active pocket of the MAO-B

**Figure 13:**
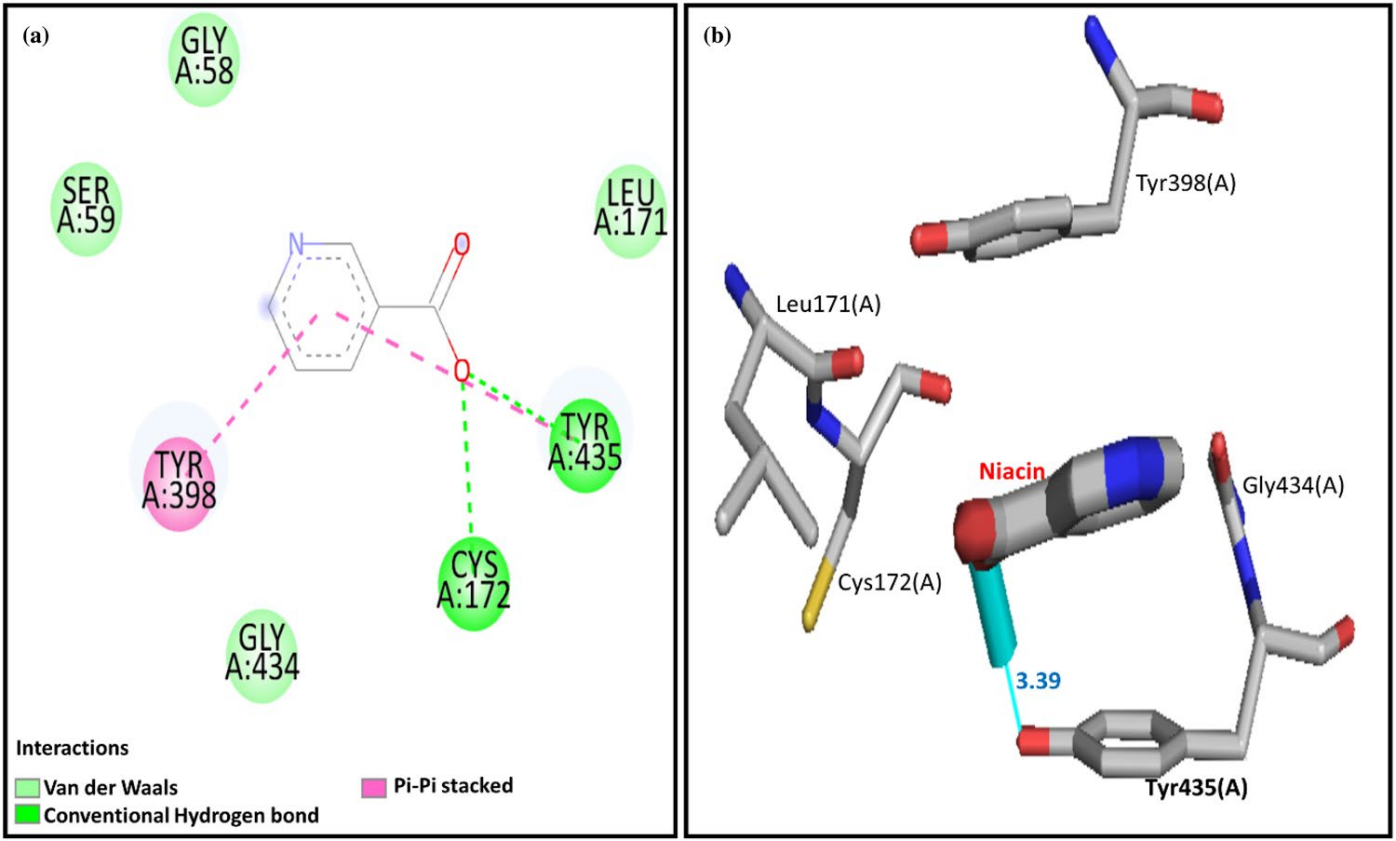
(**a**) two-dimensional, and (**b**) three-dimensional interactions between niacin and active site residues of rat monoamine oxidase B.

The binding affinity between the Niacin and the active site of MAO-B was − 5.0 kcal/mol. The interaction analysis demonstrated that the Niacin made two hydrogen bonds with active pocket residues, i.e., Y435 and C172 of MAO-B (Fig. 13a). Y435 made a hydrogen bond of the length of 3.39 Å, while the C172 hydrogen bond was not observed because of its greater length in the Ligplot analysis (Fig. 13b). Y398 made a π–π stacked bond with the Niacin. G58, S59, L171, and G434 made van der Waals interactions with the Niacin and stabilized the docked complex (Fig. 13a). The residues, i.e., Y398, G58, S59, L171, and G434, showed hydrophobic bonds (Fig. 13b).

### Effect of niacin on brain histopathology in ND ad HFD fed rats

**Figure 14:**
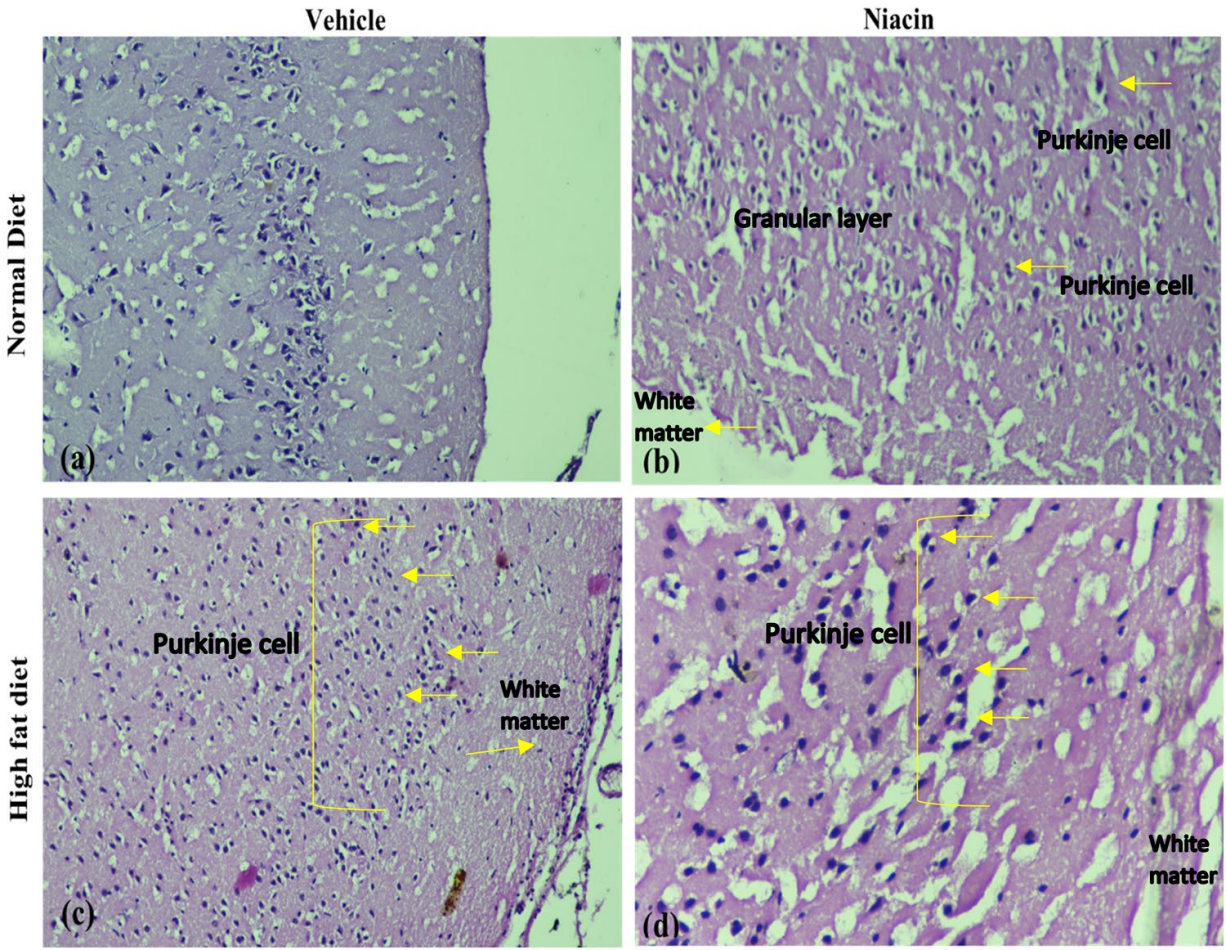
Microscopic representation of H and E staining histopathological changes at 400× in the brain of Niacin treated rats.

Figure 14 illustrates the microscopic histopathological examination of rat brain. Figure 14a depicts the typical nuclear membrane and neuronal pattern and a normal picture of nuclei. Figure 14b showed normal histology of the whole brain. Niacin administration increases the purkinje cells population. The granular cells layer contains neuronal body concentration from medium to the moderate. The white matter area was less with necrotic changes. Figure 14c showed that there were congestive changes in the brain region, the purkinje cells and granular cells were depleted that was indicated by less crowded cells possessing darkly stained characters. There was shrinkage in the granular cells and white matter contains vacuolation. Figure 14d Administration of Niacin in HFD fed animal’s normalized histology of brain. It was observed that quantity of purkinje cell increased with granular cells. The white matter area was very less with necrotic changes. It was observed that HFD had negative effect on the histology of brain while, Niacin has preventive effect on HFD induced negative changes in histology of the brain.

## Discussion

The present study was performed to determine the effect of administration of Niacin in HFD and ND on body weight changes, cumulative food intake, exploratory activity, anxiety profile, redox status, serotonin metabolism and histopathology in rats. The treatment with Niacin decreased food intake, % growth rate and oxidative stress and increased antioxidant effects and 5-HT metabolism with enhanced exploratory activity and anxiolytic effect in HFD fed animals.

Previous studies showed that HFD increased food intake ad bod weight in human^67^ ad in experimental animals^68^. The present study is also in agreement with the previous studies. Fukuwatari et al.^69^ reported that Nicotinic acid has no significant effect on bod weight and food intake in weaning rats. Previous studies also showed that Niacin supplementation decreased body weight in young rats^70^ and enhances levels of NAD and expenditure of energy^71^. In regulation of energy metabolism and lipid metabolism NAD+/NADH plays a vital role^72^. It was observed that lacking of Niacin receptor GPR109A had developed amassing of fat in liver and increased body weight^73^. Canto et al.^71^ suggested that supplementation of Niacin increased cellular NAD+ in skeletal muscles and brown adipose tissues and decreased food intake and weight gain in obese rodent fed with HFD. In the present study administration of Niacin also decreased food intake and growth rate in both HFD and ND fed rats.

HFD increases oxidative stress by lowering the level of antioxidant enzymes in brain^5^. Due to oxidative stress, SOD, which initiate the cellular defense system, changes the superoxide anion into H_2_O_2_, simultaneously others defensive enzymes i.e. CAT and GPX changes the H_2_O_2_ into molecular oxygen and water^74^. An increase in the activity of SOD enhanced the generation of H_2_O_2_, if not neutralized by CAT and GPx, induce oxidative stress by increasing contents of MDA, a by-product of lipid peroxidation and an oxidative stress marker^75^. In this study, HFD fed animals showed a decline in brain levels of SOD, CAT, and GPx activity, but higher contents of MDA estimated than ND fed animals. Niacin has shown to have antioxidant properties^76^ and capable of scavenging ROS^77^. Administration of Niacin enhanced the activity of antioxidant enzymes SOD, CAT and GPx and reduced oxidative deterioration^78^. It is mentioned in previous studies that Niacin improves inflammatory and oxidative status in dyslipidemic subjects in clinical studies^79^ and in experimental animals^80^. In the present work total protein and GSH contents in brain also increased following Niacin treatment in HFD and ND fed animals. In the earlier studies, treatment of Niacin had increased total protein contents and GSH levels during sepsis in rats^81^ and hyperlipidemic rats^82^. It is appearing in the present study that repeated treatment with Niacin inhibits oxidative deterioration by increasing antioxidant potential and reduces food intake and body weight changes, enhances exploratory activity and produces anxiolytic effect followed by HFD intake.

The basis for the present study curtailed from our present results that rats fed on a HFD were more prone to anxiety-like behavior and exhibited less motor behavior as reported by Cole et al. (2017). In a previous study, rats fed with HFD had lower rearing frequency and total distance travelled in the open field^83^ and exhibited a perplexing motor impairment before measuring the memory performance of an animal model in open field activity^84^. The open field and the home cage activity are used to assess locomotor activity in this study. Both home cage and open field activity revealed that HFD caused motor impairments that were normalized by Niacin. Previous study showed that deficiency of vitamin k reduces the locomotor activity in rats^85^. Likewise other vitamins, i.e. Niacin also increased the motor performance observed in an open field and home cage in both ND and HFD fed animal groups. Thus, it is indicated that Niacin prevents motor behavioral deficits.

Anxiety is a well-known psychiatric disease. A previous study found that when animals were exposed to an unexpected environment, their behavior changes abruptly. In an animal model, EPM and LDA had employed as a model to assess anxiety profiles^34,38^. Fear and anxiety affect the animal's locomotor activity during the EPM and LDA test, and they tend to stay in the closed sides and dark compartment respectively^86^. There is an association found between serotonin system and behavioral deficits in HFD consumption^87^. Previous research has shown that HFD fed animals showed anxiety-like behavior^10^ with decreased 5-HT and 5-HIAA levels in brain of animals^88^. In the present study, rats fed with HFD exhibited anxiety-like behavior i.e. spent less time in the light box of LDA and open arm of EPM with decreased 5-HT metabolism and increased oxidative stress. However, in fear or anxiety condition, 5-HT contents in brain become enhanced which may be due to stimulation of 5-HT-2C receptors and desensitization of 5-HT-1A receptors that increased neuronal firing from serotonergic neuron^89^. In the same time free radical production which led oxidative stress in the rats fed with HFD may also be probable reason of anxiety. In the present work reduced 5-HT metabolism with increased oxidative stress produced anxiety-like behavior. A decrease in the metabolism of 5-HT, may be due to alteration in the status of serotonergic receptors due to increased oxidative stress could be a reason of anxiety in HFD fed animals. Previous studies have found that various vitamins (Vit C, D, and B) reduced anxiety in both animals and humans^90–92^. A recent study found that Niacin had lowered the anxiety^93^. On the other hand treatment with Niacin decreased the food intake and body weight changes, increased the time that is spent in the light box and open arm in both ND and HFD animal groups with normalization of 5-HT levels and decreased redox status. It is observed from present study that Niacin has a great potential as an antioxidant and reduced anxiety like behavior following HFD administration in rats.

It has previously been reported that HFD causes necrosis and hyperplasia in various organs, as evidenced by the presence of pyknotic nuclei^94^. In the current study, fatty changes in the size and form of purkinje cells, pyknotic nuclei, and stellate cells in HFD fed rat’s brain sections indicated necrosis. Brain tissue of ND showed no inflammation and has normal morphology. The neurons were well distributed and healthy. The HFD animals treated with Niacin also showed normal morphology as ND group exhibited. Niacin administration increased purkinje cells population. The granular cells layer contains neuronal body concentration from medium to the moderate. The white matter area was less with necrotic changes. There were congestive changes in the brain region of HFD fed rats, the purkinje cells and granular cells were depleted that was indicated by less crowded cells possessing darkly stained characters. There was shrinkage in the granular cells and white matter contains vacuolation. This study is consistent with previous study reports, which found that HFD has a deleterious impact on the histology of the brain^95^. Niacin has a beneficial effect on HFD-induced alterations in brain histology.

Notably, apart from In-vitro and In-vivo studies, an In-silico approach could be important to find more information about the mechanism of intricate biological system. Molecular docking is continuously offering the great potential in the field of computer-aided drug design^96^. Moreover, inhibition of MAO by the interaction of niacin with flavin adenine dinucleotide (FAD) coenzyme is decisive in curing the enzyme from more oxidation^97^ For more immoral prime contemplation in contradiction of MAOs, the chelating activity of ligand to FAD essential to identified by an In-silico study^98^. It is very informative to find out the complexity of biological system by In-silico study for better understanding the interaction of ligand and enzyme activity. Therefore it was foreseen that Niacin is inhibited the activity of both MAO-A and MAO-B with a binding affinity estimated as ~ − 4.5 and − 5.0 kcal/mol respectively.

## Conclusion

Surely, the present study reveals that repeated administration of Niacin increases exploratory/motor activity and reduces anxiety. An increase in brain 5-HT contents appears to have an important part in the therapeutic activity of Niacin. Neurochemical results show a vital role of Niacin by inhibiting MAOs and increasing 5-HT levels. Simultaneously, Niacin reduces redox status by decreasing oxidative stress marker and increasing antioxidant enzyme is also in favor of its protective effect for reducing anxiety and enhancing exploratory activity. In-silico studies also recommend that Niacin instigated increase in 5-HT levels probably due to binding of Niacin with MAOs. Though, more studies are needed on the expression of MAO mRNA and measurement of potential kinetics of MAOs to further insight the mechanism and comprehend a role of 5-HT in the latent therapeutic effects of Niacin.

## Data availability 

The datasets used and/or analyzed during the current study available from the corresponding author on reasonable request.
